# Mesenchymal Chondrosarcoma of the Mandible: A Diagnostic Dilemma

**DOI:** 10.7759/cureus.85079

**Published:** 2025-05-30

**Authors:** Kamlesh Verma, Priyadarshini Guha, Swati Mishra, Vyas Narayan Shukla

**Affiliations:** 1 Surgical Oncology, Globe Health Care, Lucknow, IND; 2 Pathology, Globe Health Care, Lucknow, IND; 3 Pathology, Kalyan Singh Superspeciality Cancer Institute and Hospital, Lucknow, IND; 4 Orthopaedics, Government Medical College and Super Facility Hospital, Prayagraj, IND

**Keywords:** chondroid, malignant, mandible, mesenchymal tumor, rare

## Abstract

Mesenchymal chondrosarcomas (MCs) are rare malignant mesenchymal neoplasms of the head and neck region. They originate either primarily from preexisting cartilage or secondarily from Paget's disease and fibrous dysplasia. In the head and neck region, the larynx, maxilla, and nasal cavity are the most common sites. Mandibular chondrosarcoma is quite rare. On CT scan, it appears as a lobulated mass containing a chondroid matrix. Adequate tissue sampling is essential for accurate histopathological and immunohistochemical diagnosis to avoid misdiagnosis with its mimickers. The prognosis of MC is considered poor due to its high tendency for recurrence, either locally or through hematogenous metastasis. The lungs are the most common site for metastasis. Therefore, extensive resection is associated with fewer recurrences and a better survival rate than limited resection.

## Introduction

Mesenchymal chondrosarcoma (MC) is an uncommon malignant cartilaginous tumor, which is a slow-growing but aggressive tumor, representing 1% of all chondrosarcomas. The long bone is established as a common site of occurrence; however, rare occurrences are found in the head and neck region, accounting for 0.1% of all head and neck tumors [1]. They are known to arise either primarily from pre-existing cartilage or secondarily to Paget's disease and fibrous dysplasia [2].

In the head and neck region, the larynx, maxilla, and nasal cavity are the most common sites. Mandibular chondrosarcoma is known to be quite rare. Generally, it is not associated with regional lymphadenopathy. On CT scan, it is reported as a lobulated mass with a chondroid matrix [3]. Therefore, adequate biopsy is required to identify features necessary for accurate diagnosis and to avoid misdiagnosis of Ewing family tumor, chondromyxoid fibroma, and other mimickers [4,5].

Diagnostic histopathological features include cellular, undifferentiated round tumor cells with ovoid to spindle morphology, along with cartilaginous differentiation. Hemangiopericytoma-like vascular areas and calcification are also noted [6]. In small biopsy tissue, diagnosis is challenging; therefore, immunohistochemistry (IHC) becomes extremely important in such cases [7,8].

In this case report, we highlight the diagnostic difficulty in identifying MC and its distinction from close mimickers in biopsy specimens from rare sites such as the mandible.

## Case presentation

A 25-year-old man presented to the oncosurgery unit of our hospital with a complaint of gradually progressive swelling in the left parotid region for one year, associated with weight loss and difficulty in swallowing. On clinical examination, a large, hard mass with smooth margins measuring 4 × 5 cm was noted. There was no significant past history. A CT scan showed a heterogeneously enhancing soft tissue space-occupying lesion measuring 4.7 × 4.2 × 3.7 cm in the left parotid region with calcification.

**Figure 1:**
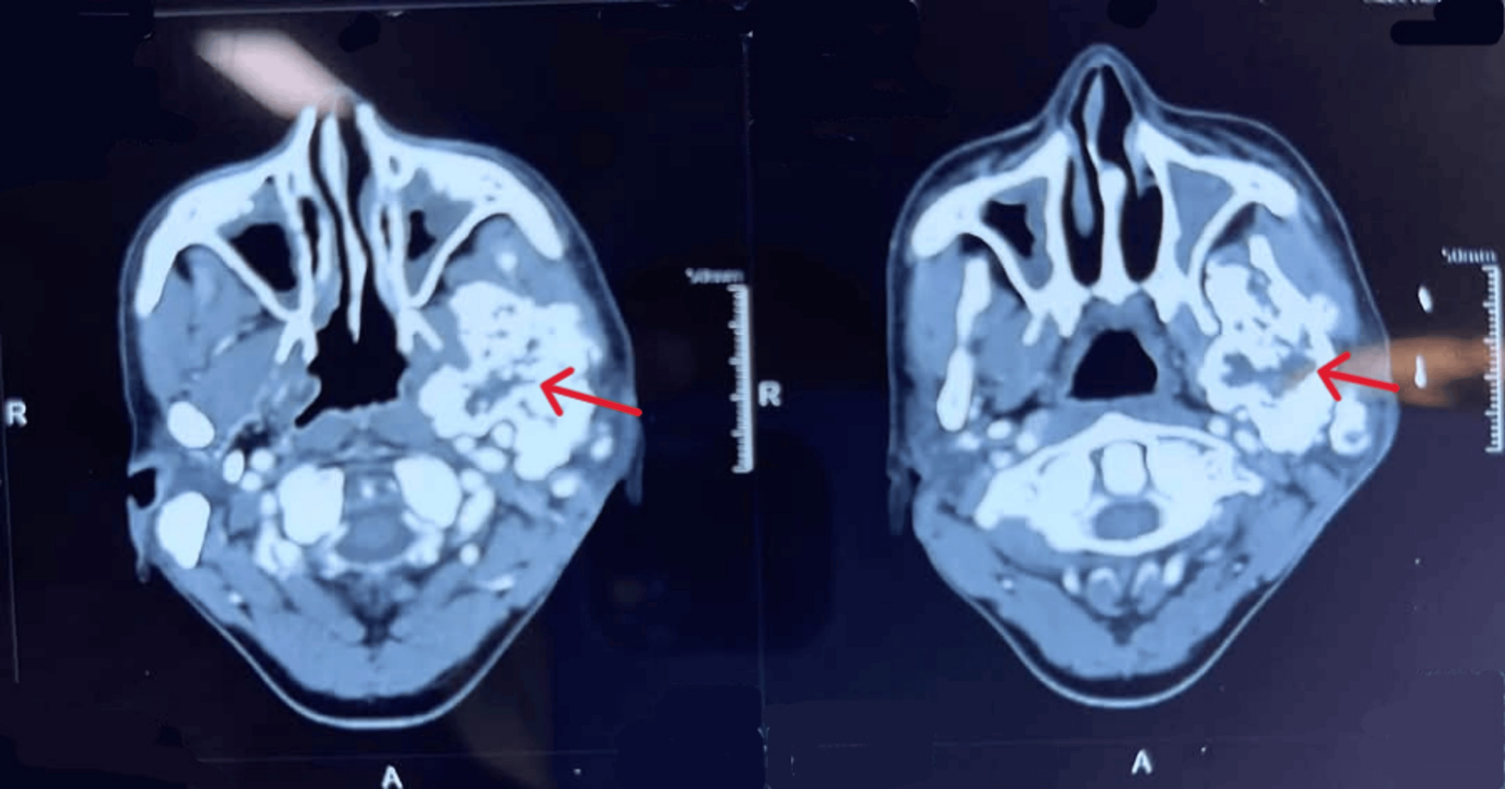
Positron emission tomography–contrast-enhanced computed tomography A non-FDG avid lytic expansile lesion in the left ramus and coronoid process of the mandible (size: 5.1 x 4.3 x 6.3 cm) (red arrow). FDG: fluorodeoxyglucose.

At our hospital, PET-CECT (positron emission tomography-contrast-enhanced computed tomography) was performed, which revealed a non-FDG-avid lytic expansile lesion in the left ramus and coronoid process of the mandible (size: 5.1 × 4.3 × 6.3 cm), with a non-FDG-avid calcified lesion/extension measuring 1.9 × 3 cm (Figure 1).

**Figure 2:**
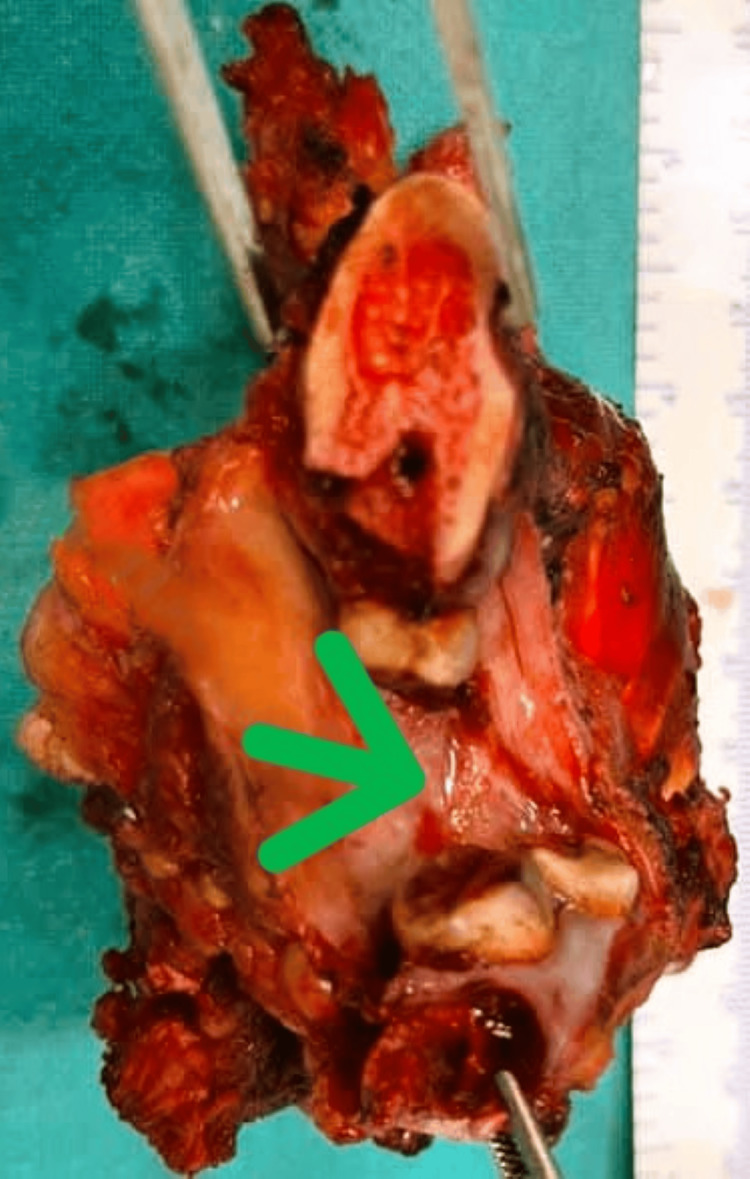
Gross image A lobulated tumor in the ramus and coronoid process of the mandible (green arrow).

The patient underwent left bite composite resection following a final diagnosis of MC on biopsy. The surgery was uneventful, and the specimen was sent for histopathological examination. Grossly, the tumor appeared grayish-blue and lobulated, located in the ramus and coronoid process of the mandible, measuring 7.5 × 4 × 3.5 cm (Figure 2). The cut surface of the tumor was glistening. The mandible was involved by the tumor, sparing the maxillary bone.

**Figure 3:**
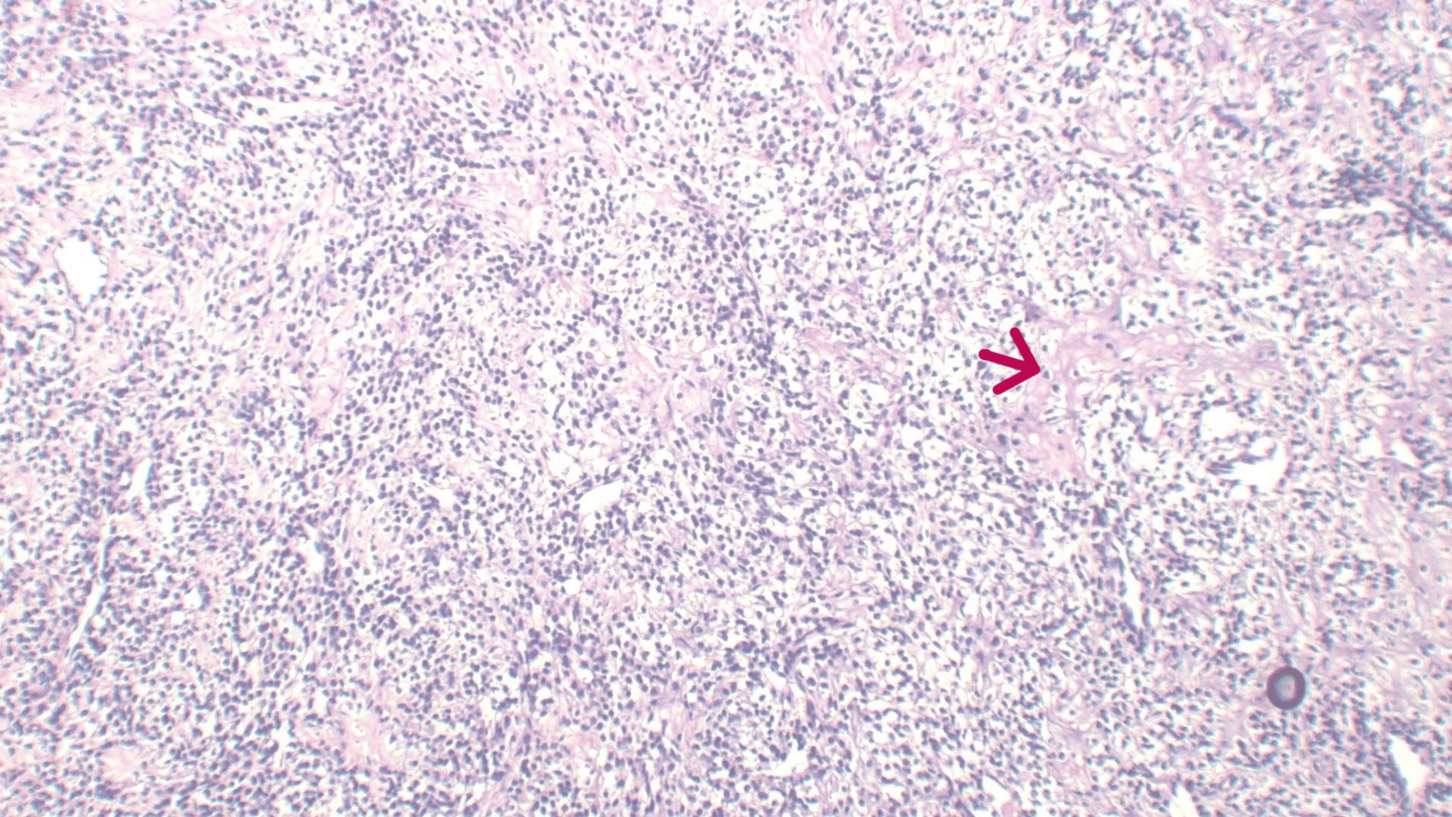
Hematoxylin and eosin (H&E) stain, 40X magnification Sheets of small round tumor cells with fine to coarse chromatin, inconspicuous nucleoli, and scant to moderate clear cytoplasm. Perivascular arrangement of tumor along with bits of mature cartilage (red arrow).

Microscopy of the resected specimen showed sheets of small round tumor cells with fine to coarse chromatin, inconspicuous nucleoli, and scant to moderate clear cytoplasm. Perivascular arrangement of tumor cells was also noted, along with bits of mature cartilage (Figure 3). All resection margins were free of tumor invasion.

**Figure 4:**
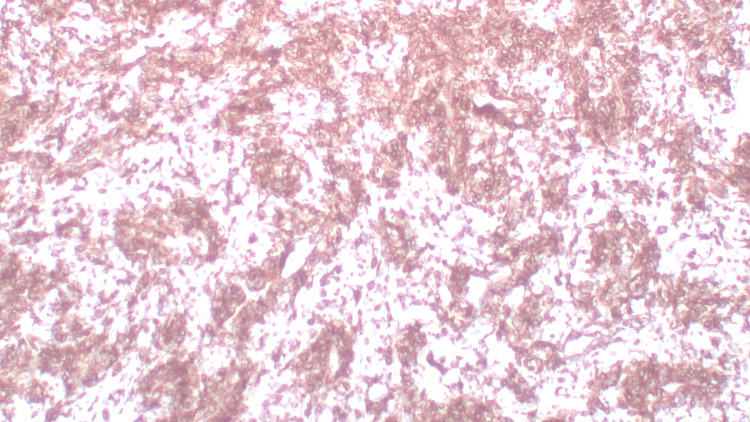
Immunohistochemistry (IHC) for CD99 (40×) Tumor cells show positive immunoreactivity for CD99.

**Figure 5:**
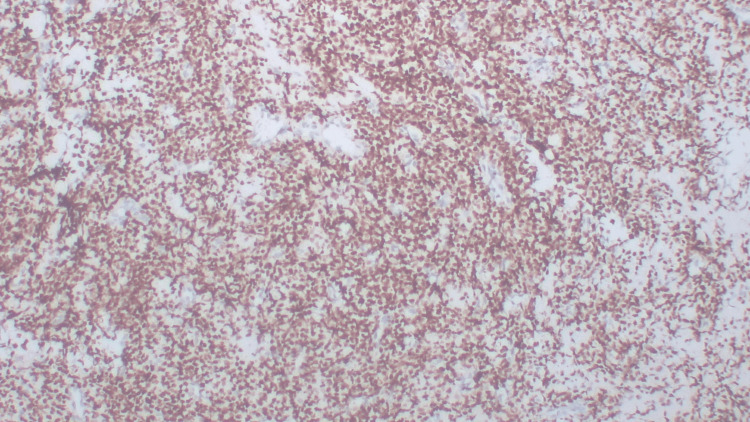
Immunohistochemistry (IHC) for NKX2.2 (40×) Tumor cells show positive immunoreactivity for NKX2.2.

**Figure 6:**
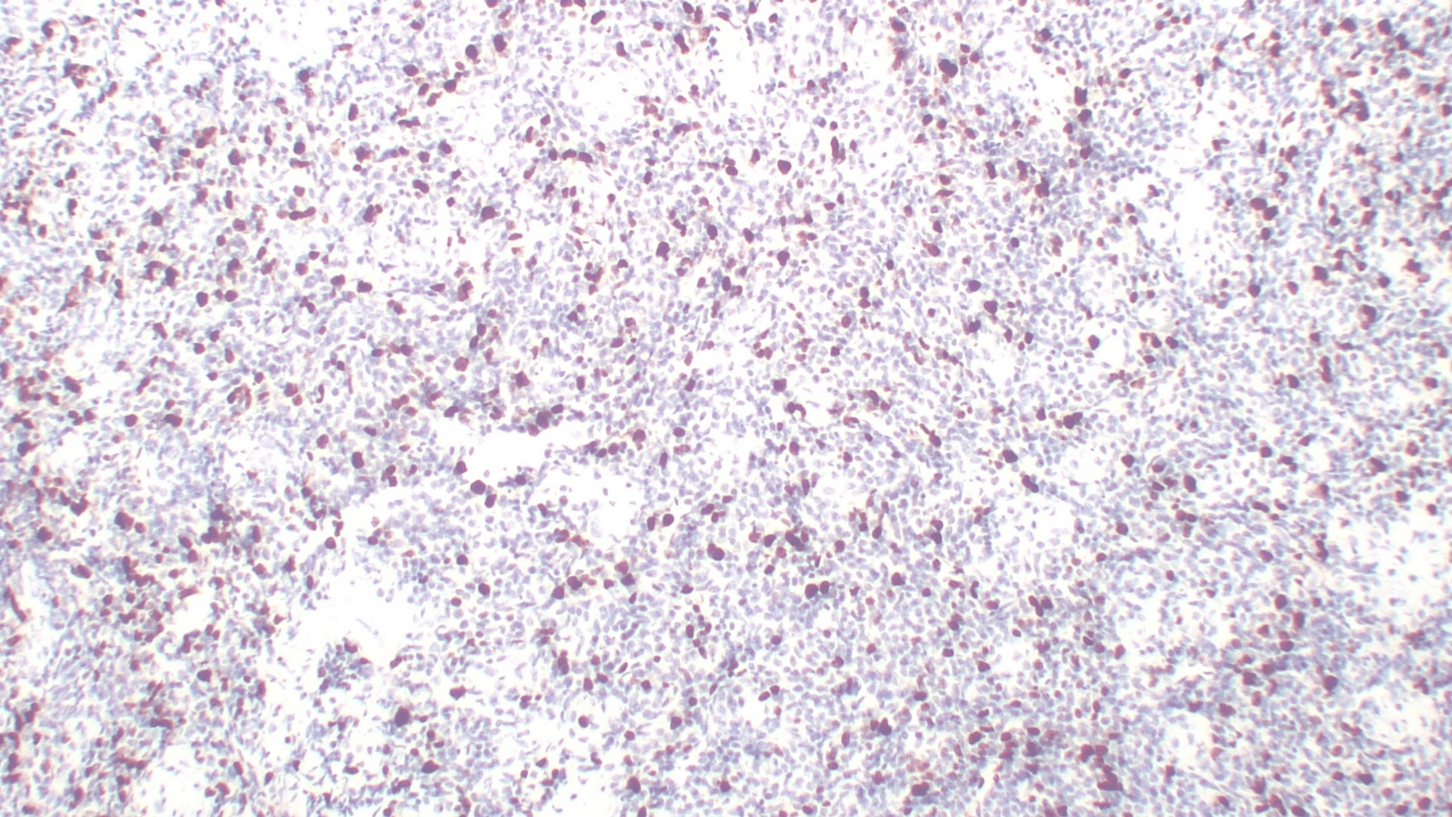
Immunohistochemistry (IHC) for Ki-67 (40×) Tumor cells show positive nuclear immunoreactivity for Ki-67 (~40%).

On immunohistochemistry, the tumor cells showed positivity for CD99 (Figure 4), NKX2.2 (Figure 5), Ki-67 (~40%) (Figure 6), and NKX3.1. The tumor cells were negative for S100, SMA, CK, CD45, Desmin, and WT-1.

Based on histopathology and immunohistochemistry, a diagnosis of MC was made. A follow-up after two months showed no signs of local recurrence in the patient.

## Discussion

MCs are rare malignant mesenchymal neoplasms, particularly in the head and neck region. They arise either primarily from pre-existing cartilage or secondarily due to Paget's disease and fibrous dysplasia [2]. In the head and neck region, the larynx, maxilla, and nasal cavity are regarded as the most common sites. Mandibular chondrosarcoma is observed quite rarely. Generally, it is not associated with regional lymphadenopathy. On CT scan, it appears as a lobulated mass with a chondroid matrix [3]. Adequate tissue sampling is therefore of utmost importance for accurate histopathological diagnosis [4]. Inadequate biopsy will usually lead to misdiagnosis with Ewing family tumors, chondromyxoid fibroma, and other mimickers [5]. Diagnostic histopathological features required for an accurate diagnosis include cellular, undifferentiated round tumor cells displaying ovoid to spindle morphology, along with cartilaginous differentiation. Hemangiopericytoma-like vascular areas and calcification are also noted [6].

In small biopsy tissue, diagnosis is challenging. IHC is helpful in such scenarios. S-100 is strongly positive in conventional chondrosarcoma; however, in the round cell component of MC, it shows loss of expression. Type II collagen is designated as a specific marker for MC [7,8]. Histopathological mimickers like Ewing’s sarcoma show similar histological features but differ by lacking cartilaginous areas and the hemangiopericytoma-like pattern [9]. CD99 positivity is seen in both MC and Ewing’s sarcoma, along with vimentin, CK, NKX2.2, FLI1, and CD99 [10]. Another differential is angiosarcoma, due to the hemangiopericytoma-like pattern perceived in MC. However, angiosarcoma lacks cartilaginous differentiation and displays positivity for CD31 and CD34. Synovial sarcoma is also a differential, as it shares similar histology, but S-100 positivity and CK negativity help in distinguishing these tumors. The presence of a lace-like osteoid matrix in chondroblastic osteosarcomas helps differentiate them from MC [11].

Although there is variation in opinion regarding the standard treatment protocol for MC [12], the most effective therapeutic modality recognized is wide surgical excision followed by chemotherapy and radiotherapy, if needed [13]. In the case of mandibular involvement, a wide local excision with a tumor-free margin of 2 to 3 cm is recommended. Extensive resection is associated with lower recurrence and better survival rates compared to limited surgical resection [14]. The prognosis of MC is poor because of its high tendency for late recurrences, either locally or via hematogenous metastasis [15]. The lungs are considered the most common site for metastasis. Five-year survival rates have been reported for craniofacial MC [16,17].

## Conclusions

Mesenchymal chondrosarcomas are considered aggressive cartilaginous tumors in the head and neck region. In the head and neck region, the larynx, maxilla, and nasal cavity are found to be the most common sites. Chondrosarcoma of the mandible is quite rare. The biphasic appearance of the tumor, in the form of small round blue cells admixed with mature cartilage, creates a diagnostic challenge at such rare sites.

Our study highlights the importance of obtaining an adequate biopsy, along with meticulous histopathological examination and detailed immunohistochemical analysis, for definitive diagnosis and differentiation from close potential mimickers such as Ewing sarcoma, chondromyxoid fibroma, and angiosarcoma. Given the high recurrence rate and metastatic potential, accurate diagnosis is crucial to enable proper resection by surgeons and to provide a high disease-free survival rate. Therefore, awareness of this rare mandibular disease should be considered when evaluating lesions at this unusual location. Through the use of histopathology and immunohistochemistry, accurate diagnosis can be proffered.
